# The effect of radiofrequency heat treatment on trypsin inhibitor activity and in vitro digestibility of soybean varieties (*Glycine max.* (L.) Merr.)

**DOI:** 10.1007/s13197-022-05523-z

**Published:** 2022-06-29

**Authors:** Krisztina Takács, Erika E. Szabó, András Nagy, Zsuzsanna Cserhalmi, János Falusi, Éva Gelencsér

**Affiliations:** 1 Institute of Food Science and Technology, Department of Nutrition Science, Hungarian University of Agriculture and Life Sciences, Somlói Road 14-16, 1118 Budapest, Hungary; 2 Cereal Research Non-Profit Ltd, Alsókikötősor 9, 6726 Szeged, Hungary

**Keywords:** Trypsin inhibitor, Radiofrequency heat treatment, In vitro digestibility, Native-PAGE

## Abstract

Kunitz (KTI) and Bowman-Birk (BBI) trypsin inhibitors were characterized in soybean seeds. Cultivars having KTI/BBI (Pannónia Kincse, PK) or lacking KTI (Aries; Hilario; Bahia) were assessed with well-characterized soybean varieties having Ti-a or ti types of KTI mobility. The TIA values of Pannónia Kincse (9.8 ± 0.48 mg/g) were not significantly different (p ≤ 0.05) from Ti-a samples (10.07 ± 1.86 mg/g), while of Aires, Bahia, Hilario (6.19 ± 1.89) were identical (p ≤ 0.05) with ti samples (6.63 ± 1.99). Radiofrequency heat treatment (RF) decreased TIA values (p ≤ 0.05) at ≥ 100 °C. However, in the traditional soybean variety, the RF at 110 °C was more effective in eliminating the residual KTI activity. The remaining or the disapperaing bioactive form of trypsin inhibitors were succesfully characterized by the means of a standardized in vitro digestion model. It showed that residual BBI-originated trypsin inhibitor activity was in the stomach even after RF at 110 °C, whereas its chymotrypsin inhibitor activity was not detectable at all. Although PK and KTI null types of soybean seeds still required an energy-saving, gentle heat treatment to inactive the trypsin inhibitors before using them as food or feed, the physicochemical properties and processing quality of soybean products were protected, improved.

## Introduction

The major protease inhibitors in soybean belong to Kunitz (KTI) and Bowman-Birk (BBI) types of inhibitor families. These antinutritive compounds inhibit the dietary protein digestion, and the absorption, due to the formation of complexes, that are resistant to the intestinal digestion, and even if the diet consists of free amino acids reduces the growth performance of the animals. It was also found, that KTI and BBI induce enlargement of the pancreas and hypersecretion of digestive enzymes in rodents and birds. As soybean is deficient in sulphur containing amino acids, as methionine, the loss of large amounts of such sulphur-rich endogenous proteases leads to the weight depression (Lajolo and Genovese 2002).

KTI mainly inhibits specifically the trypsin like proteases and weakly inhibits chymotrypsin. KTI is responsible for the whole soy trypsin inhibitor activity in 60%. Long-term consumption of active KTI at a high level reduces protein digestibility and cause pancreatic carcinogenesis (Vagadia et al. 2018).

Soybean BBI inhibit potently and specifically trypsin- and chymotrypsin-like proteases simultaneously, but independently two, either the same or different, enzymes, thus trypsin/trypsin, trypsin/chymotrypsin. While amino acid sequence variation within N-terminal inhibitory domain profoundly affects inhibitory potency against trypsin, C-terminal provide specificity against trypsin- or chymotrypsin-like enzymes (He et al. 2017). Physiologically relevant amounts of BBI type inhibitors are resistant in the gut and can reach the large intestine in active form due to their structural and functional stability. However, some studies showed that BBI appeared to be universal cancer preventive agent and a potential candidate in prevention of colorectal cancer. Although the mechanism of this activity has not been elucidated, there is considerable evidence suggesting that BBI exert their preventive properties via protease inhibition (Clemente and Arques 2014).

It was shown that elimination of inhibitory activity significantly increases the dietary protein utilization. Despite of the most commonly used thermal treatments, such as hot-air drying and roasting effectively inactivate the antinutritional factors in soybean, usually require extremely long exposure time (10–20 min) at high temperature (100–200 °C) due to the high thermo-stability of trypsin inhibitors (Jiang et al. 2020). The prolonged heating induces a surface overheating and dehydratation thereby reducing the storage stability of the final products. Although the prolonged heat treatment effectively inactivates trypsin inhibitors, but it denatures soybean proteins resulting degradation of essential amino acids and makes impared utilization of oxidated sulphur-containing amino acids or non-reactive lysine formation and leading to other deteriorative reactions. It has a great influence not only on the nutritional value of soy flour but also on the sensory and functional properties depending on the type and intensity of heating (Khattab and Arntfield 2009).

To minimise the unfavourable effects, moderate heat treatment techniques can be used, such as infrared treatment (Yalcin and Basman 2015), controlled instantaneous pressure drop technique (Détente Instantane´e Contrȏlée, DIC) (Haddad and Allaf 2007), microwave and the high hydrostatic pressure (HHP) treatments (Zhong et al. 2015), and some others. They offer not only a potential for fast and constant heating rate compared to common heating methods but also have a beneficial effect on the nutritional value via partial denaturation of soy proteins facilitating easier enzyme access and thereby an improved protein digestibility. These types of processing are energy efficient (environment-friendly) have lower production costs (Vagadia et al. 2018). At the same time, problems concerning the surface overheating and dehydratization in large volume products still exist due to their poor penetration depth (Jiang et al. 2020).

Recent studies have reported that RF treatment with bulk heating and greater penetration depth can overcome the deleterious effects caused by other heating methods (Jiang et al. 2020). It is a novel heating technique based on capacitive dielectric heating. It can rapidly deliver energy uniformly within the food material at lower frequencies than microwave and infrared heating, and thus enabling a better penetration depth, which makes it suitable to avoid surface overheating and dehydration.

The principles of RF heating are discussed in details by Ling et al. (2019), but very few information is still available on its effect on the core seed protein structure, so further studies are needed in this field. The successful application of RF depends on factors as the nature of the dielectric material including structure and moisture content, condition when dielectric heating applied (frequency, density, temperature) (Hassana et al. 2019). Some studies have described that RF treatment could effectively be used for inactivation the trypsin inhibitors in soaked soybean (Zhong et al. 2015). Yet, this method was not economically viable in soybean processing because this treatment was associated with the generation of considerable quantities of waste- water, which resulted in a short shelf life induced by bacteria. Furthermore, the potential cell damage could cause also nutrient losses by interacting of released antinutrients with different endogenous substrates. Therefore, a novel method was intended to be used for inactivating antinutrients in intact soybean with low moisture content. Physicochemical properties and processing quality of soybean products has been improved by RF heating when trypsin inhibitor activity was inactivated within 300 s resulting 89.4% inactivation rates, while the conventional thermal treatment significantly decreased functional properties and led to the formation of greater aggregates (Jiang et al. 2020). During RF treatment, the polar groups in protein molecule are capable to absorb more waves by heating and generate free radicals, which might lead to aggregation and unfolding of the protein. The interactions of these free radicals among the protein molecules may cause modifications in the protein formation and its functional properties (Cerny and Hobza 2007). Guo et al. (2017) showed that treatment of soy protein isolates (SPIs) with RF waves under different temperature up to 90 °C did not modify the primary structure of the protein. RF heating induced significant increase in free sulfhydryl groups and surface hydrophobicity in secondary and tertiary structures of SPIs.

The effect of heating on trypsin—and chymotrypsin inhibitor activity (TIA/CIA), inactivation might be closely correlated with the structures of KTI and BBI. When soymilk was treated using a conventional heating method, KTI’s TIA could be rapidly inactivated by incorporation of KTI into heat-induced protein aggregates through non-covalent interactions and/or SS bonds (the products of SH/SS exchange and SH oxidation reactions), and KTI’s TIA loss was positively correlated with soymilk protein aggregate amount (Xu et al. 2012). As in KTI only two disulphide bonds link the monomers, the 3D structure is unstable, so thermal inactivation of KTI occurs rapidly leading aggregated forms, and thus KTI becomes inactive for trypsin due to the confirmational change.

BBI’s TIA and CIA were simultaneously slowly inactivated by heat-induced cleavage of 1 BBI peptide bond. So confirmational changes occur and BBI becomes inactive for chymotrypsin. BBI and its fragments did not tend to interact with other proteins due to its highly hydrophilic nature, as BBI was composed of 75% hydrophilic amino acid residues (Lu et al. 2015).

These results suggested: RF heating has a great potential in effective inactivation of the residual BBI activity in intact soybean, which is still an existing problem and could be a candidate to displace the conventional thermal treatment in soybean industry. It was reported: approximately 60% of BBI inactivation was required for the highest nutritive value of soybean, which might be correlated with a heat-induced sulfhydryl/disulphide exchange reaction, protein fragmentation, formation of protein aggregates or Maillard reaction (He et al. 2017).

The cost of heat treatment can be saved, and sustainability improved by using reduced KTI TI or ti null types of soybean varieties. However, it was not examined whether the de-oiled protein rich soybean products of these soybean varieties are suitable for direct feed or food use concerning their protease inhibitor activity or it would be necessary to use an energy-saving, gentle process (He et al. 2017).

We characterized the main protease inhibitor profile in high-yielding traditional and the KTI-null types of soybeans, and the temperature effect on the residual TIA to obtain an improved protein digestibility after a low frequency and short time exposure of RF treatments. Furthermore, we simulated digestion to monitor its effect on the TIs bioactivity and bioaccessibility.

## Materials and methods

### Soybean samples

Glycine max (L) Merr. soybean seeds involved into the domestic cultivation in Hungary were provided by the Cereal Research Non-Profit Ltd., Szeged, Hungary (CR Ltd.) in two subsequent years, such as the high yielding, convenventional Pannónia Kincse (HU 149,567; PK) developed by CR Ltd, and a range of newly developed by Società Italiana Sementi, Italy (SIS) soybean varieties with low anti-nutritional factor content, Aires (IT 224, A), Hilario (H), Bahia (IT 224, B), registered in EU Plant variety database (https://ec.europa.eu/food/plants/plant-reproductive-material/plant-variety-catalogues-databases-information-systems_en Accessed on 10th May, 2022). KTI-null phenotypes carrying ti allele (PI 542,044, PI 547,816, PI591539, PI547656, PI 547,660, PI 157,440, PI 196,168), conventional phenotypes carrying Ti-a (PI 54,807, PI 548,533, PI 548,532, PI 548,573, PI 548,631, PI 518,671) from USDA-ARS Soybean Germplasm Collection (http://grscicoll.org/institutional-collection/usda-soybean-germplasm-collection, Accessed on 10th May, 2022) were also provided as references. Seeds were directly used for RF treatments. For molecular analyses, seeds were necessary to be milled (0.5 mm, Hauser G-742 grinder).

### Detection of KTI presence or absence by PCR

Wizard® DNA Clean-up System was applied to isolate DNA according to the technical bulletin. 860 μl of Wizard buffer (pH 8.0), 100 µl of 5 M guanidine hydrochloride, 40 µl of proteinase K enzyme (20 mg/ml) were added to 100 μg defatted, milled soybean varieties (PK, A, B, H), homogenized, rotarily incubated (3 h, 60 °C), centrifuged (12,000 rpm, 10 min). 500 μl of pure supernatant was mixed to 1 ml of Wizard minipreps DNA Purification Resin (Promega), and passed through a DNA-binding minicolumn (resin) using a syringe. Then, the column was washed with 2 ml of 80% isopropyl alcohol, followed by the elution of DNA with 70 °C Tris–EDTA buffer (pH 7.6), centrifugation (12,000 rpm, 2 min). Concentration and purity of the isolated DNA were measured at Abs260/280 nm in one step.

DNA sequences were amplified in a reaction mixture of 25 μl containing: 30 ng of DNA and 0.5 μM of specific primer pairs (Biometra TOne gradient PCR instrument). The presence of dominant TiTi gene (1 allele) or recessive titi gene (2 allele) was determinated by Satt228 primer pairs (Forward primer sequence: 5′-TCATAACGTAAGAGATGGTAAAACT-3′, Reverse primer sequence: 3′- CATTATAAGAAAACGTGCTAAAAGAG-5′) developed by Kim et al. (2006). PCR reaction’s condition: Steps: 1. Initial denaturation 95 °C, 30 s; 2. Denaturation 95 °C, 30 s; 3. Annealing 62 °C, 45 s; 4. Extension 72 °C, 30 s; 5. Final extension 72 °C, 180 s; where 1. Cycle No.: 1; 2–4: Cycle No.:34; 5. Cycle No.:1

PCR results were evaluated by FlashGel™ System.

### Determination of trypsin inhibitor activity

Soybean flour (1 g) in 50 ml of 0.01 M NaOH (pH 9.5) was extracted (2 h, constant stirring, 25 °C); allowed to stand (overnight, 4 °C); further diluted with 50 ml of cold distilled water (4 °C) by shaking; own-sedimented (15 min). The collected supernatant was diluted in distilled water to a content of 40–50% TIA. 1 ml of that mixed with 5 ml of L-BAPA (Na-benzoyl-DL-arginine 4-nitroanilide hydrochloride, 60 mg/1 ml DMSO made up to 100 ml with 0.005 M CaCl_2_*2H_2_O buffer solution (pH 3.0) and 2 ml of distilled water, then incubated for 10 min at 37 °C. Then 1 ml trypsin (stock: 27 mg trypsin/100 ml CaCl_2_*2H_2_O buffer solution (pH 3.0), 5 ml of stock was made up to 100 ml same buffer) was added, incubated (37 °C, 10 min). Reaction was stopped by 1 ml 5.3 M acetic acid. Mixture was finally centrifuged (3800 rpm, 15 min). The quantity of released *p*-nitroaniline is measured at 410 nm; calculated in terms pure trypsin/g sample as weighed (mg/g) according to ISO 14902:^2001^.

### Separation of proteins by native (non-denaturing conditions) polyacrylamide gel electrophoresis and identification of trypsin and chymotrypsin inhibitor activity

3 mg soybean flour was extracted in 60 μl sample buffer (sample buffer: 800 mg saccharose in 4 ml running buffer). Electrophoresis -in the absence of SDS in gel and running buffer- was performed following the Instruction Manual of Mini-PROTEAN 3Cell (BioRad) using 6%/12% stacking/resolving gel, Laemmli sample buffer, Tris–glycine containing running buffer (Laemmli 1970).

In case of activity visualization, TIA in the gels was shown by reaction with trypsin or chymotrypsin and its substrate, N-acetyl-DL-phenylalanine β-naphthyl ester, followed by negative staining with tetrazotized 0′-dianisidine-ZnCl_2_ complex (Uriel and Berges, 1968). Finally, 0,2% Coomassie Brilliant Blue R-250 dye was used. Staining Image analyses of gels were carried out with BIO-RAD Gel Doc 2000 system, and evaluated by BIO-RAD Quantity One 4.3 version.

### Radio-frequency heat treatment

Ti (PK) and ti-type (A) of soybean variants from the second year of the experiments were selected to monitor the RF-effect on the bioactivity of TIs. Bulk of soybean seed (~ 5 kg) was premoistured to 20.3% by tap water, then was warmed up to 75, 100, 110 °C—measured with three fiber optic thermometers in three different locations—in the teflon plate by RF generator (Brown Boveri, Switzerland) with 10 kW power on 13.5 MHz frequency, for 50–90 s. After treatment, seed was cooled to ambient temperature.

### In vitro digestion of soybean samples

The resistance of proteinase inhibitors of RF treated soybean flours (PK, A) to gut digestion process was assessed in human in vitro static digestion model Minekus et al. (2014). Non-heat-treated samples and purified KTI and BBI were applied as internal controls.

Simulated oral (pH 7.0), gastric (pH 3.0), and intestinal (pH 7.0) digestion fluids were adjusted according to the assumable physiological circumstances: human salivary alpha amylase 75 U/ml in the final oral bolus, pepsin 2000 U/ml in the final gastric bolus, pancreatin 100 TAME U/ml—based on trypsin—and bile extract (10 mM) in the final intestinal bolus was added additionally to the intestinal bolus. Oral, gastric and intestinal phases were done at 37 °C for 2 min, 2–2 h, respectively.

### Statistical analysis

Each experiment was performed in triplicate with parallel samples. The results were presented as mean ± standard deviations (SD). Treatment comparisons were evaluated by one-way analysis of variance (ANOVA). Differences were considered statistically significant at P < 0.05. Statistical tests were performed using IBM SPSS Statistics 22 Software.

## Results and discussion

**Fig. 1:**
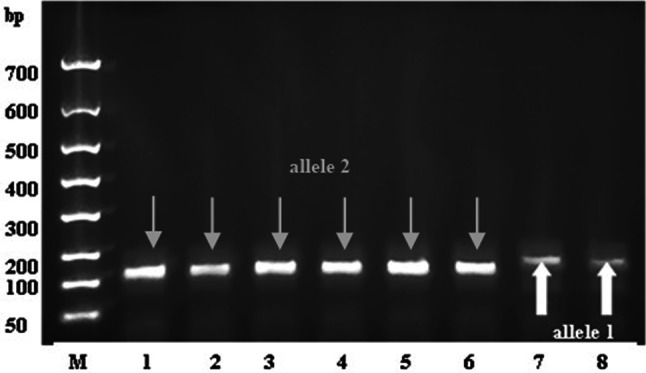
Patterns of amplified DNA fragments for Satt228 in conventional soybean variety (PK) and new soybean varieties with low trypsin inhibitor contents (A, B, H). PK amplified allele 1 (TiTi gene, lanes 7–8) and A, B, H amplified allele 2 (titi gene, lanes 1–6) from the genomic DNA with the Satt228 marker by PCR. Lanes: M: Marker, 1–2. Aires (A); 3–4. Bachia (B); 5–6. Hilario (H); 7–8. Pannónia Kincse (PK)

PCR technique was applied to be proved the presence of KTI Ti-a and ti alleles in the soybean varieties. Kim et al. (2006) identified that microsatellites or simple sequence repeats (SSR) marker-specific Satt228 DNA marker tightly linked to the Ti locus which was responsible for controlling of the presence of the KTI mobility or KTI absent mobility variant proteins. In the presence of a dominant KTI gene (TiTi gene), allele 1, while in the KTI absent mobility lines (recessive titi gene), allele 2 gave a signal by gelelectrophoresis. In Fig. 1. we have demonstrated that allele 1 (TiTi gene, ~ 180 bp, Lanes 7, 8) was amplified from the genomic DNA by PK, while allele 2 (titi gene, ~ 160 bp, Lanes 1–6) by A, B, H varieties.

**Fig. 2:**
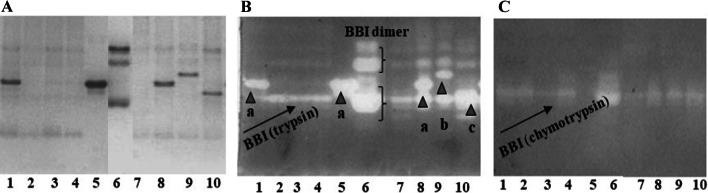
Protease inhibitor patterns of soybean proteins [*Glycine max.* (L.) Merr.] in high yielding, conventional (PK) and new soybean varieties with low trypsin inhibitor contents (A, B, H) and well characterized KTI mobility (Ti-a) and KTI absent (ti) mobility types of soybean varieties. The purified proteinase inhibitor proteins (KTI, BBI) were used as inside controls. Proteins were separated in 6/12% native-PAGE gel **a** than the gels were treated with trypsin **b** or chymotrypsin **c** using the negative enzyme staining method to be identified the KTI or BBI originated inhibitory activity. Lanes: 1. Pannónia Kincse (Ti-a), 2. Aries (ti), 3. Bahia (ti), 4. Hilario (ti), 5. KTI (Sigma T2327), 6. BBI (Sigma T9777), 7. PI 157,440 (ti), 8. PI 518,671 (Ti-a), 9. PI 547,891 (Ti-b), 10. PI 86,084 (Ti-c)

The main proteinase inhibitor profiles of novel soybean varieties were assessed by 6/12% native-PAGE separation (Fig. 2a) in comparison with well characterized soybean varieties of KTI (Ti-a) and KTI absent (ti) mobility types. Electrophoretic patterns were not different in a view of the two subsequent years of cultivation, the presence and distribution of proteins were the same, so representative gel photos are presented**.** Since KTI and BBI are responsible for a significant part of the enzyme inhibition in soybean varieties carrying the KTI mobility protein, and BBI is responsible for the enzyme inhibition in the KTI-absent soybean variants, the purified KTI (Lane 5) and BBI (Lane 6) proteins were used as internal standards. The KTI has appeared in one distinct band, whereas BBI was displayed in three distinct bands. Pesic et al. (2007) have published similar results and gave such an explanation that the zone with lower electrophoretic mobility (I) represents the polymeric forms of BBI, whereas the other (II) represents monomeric form of BBI. The presence of polymeric forms of BBI is a result of their self-aggregation under non-dissociating conditions. The trypsin inhibitor profile of the traditional soybean variety (PK, Lane 1) amplifying the allele 1 was comparable with the KTI Ti-a mobility type of soybean variety (PI 518,671, Lane 8), whereas the low trypsin inhibitor activity soybean varieties (A, B, H, Lanes 2, 3, 4 respectively) which amplified the alleles 2 gave similar protein pattern to the KTI free mobility soybean variant (PI 157,440, Lane 7). It was demonstrated that PK belonged to the KTI mobility phenotype carrying the high frequency of Ti-a allele (Lee et al. 2011) characteristic also for EU cultivars, whereas soybean cultivars A, B, and H matched the KTI-absent mobility soybean phenotype carrying the ti allele.

Specific negative enzyme staining methods were applied for identification their KTI and BBI originated trypsin and BBI originated chymotrypsin inhibition activities**.**

Trypsin active bands (Fig. 2b) were identified in the KTI (Lane 5) and in the two main zones (I, II) of BBI (Lane 6). Both KTI- and BBI-derived trypsin active signals were detected in the KTI mobility soybean varieties (PK, Lane 1; PI 518,671, Lane 8), whereas BBI-derived trypsin inhibitory activities were proved in the KTI absent soybean phenotypes (A, B, H, Lanes 2, 3, 4 respectively; PI 157,440, Lane 7).

The alpha-chymotrypsin specific activity (Fig. 2c) was confirmed in the monomeric form of BBI (Lane 6). BBI derived chymotrypsin active signal was obtained in both soybean phenotypes carrying Ti (PK, Lane 1; PI 518,671, Lane 8) and ti alleles (A, B, H, Lanes 2, 3, 4 respectively; PI 157,440, Lane 7).

**Fig. 3:**
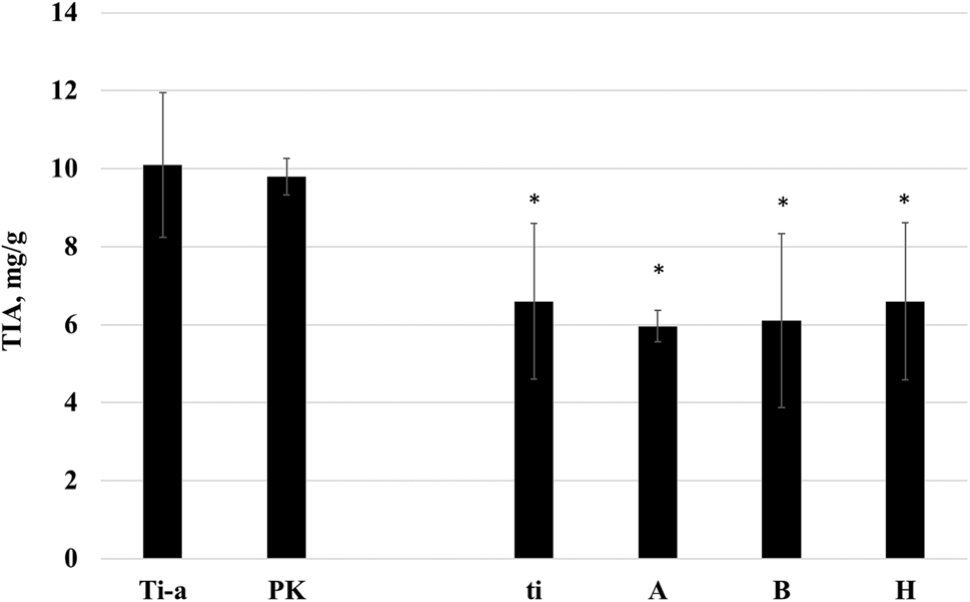
The combined results of TIA (Mean ± SD) values in soybean varieties of *Glycine max.* (L) Merr. containing the two major types (KTI, BBI) of trypsin inhibitors (PK) and a range of KTI absent variants (A, B, H) obtained from the two subsequent years of national cultivation and well characterized soybean varieties (PI 518,671, Ti-a; PI 157,440, ti). Values within each column with the * marking are significantly different from PK value (p < 0.05)

*The total trypsin inhibitor activity (TIA) values in crude meals (PK, A, B, H)* obtained from the domestic cultivation of two subsequent years did not show significant differences (p ≤ 0.05), therefore the results of the two years were combined (Fig. 3.). It was found that TIA values of crude PK samples (9.8 ± 0.48 mg/g) were not significantly different (p ≤ 0.05) from those ones obtained for Ti-a samples (10.07 ± 1.86 mg/g), whereas the TIA values for ti allele containing A, B, H samples (6.19 ± 1.89 mg/g) were identical (p ≤ 0.05) with the results obtained for ti (6.63 ± 1.99 mg/g) samples. As it was expected, the TIA values for the newly developed conventional soybean variety (PK) significantly exceeded (p ≤ 0.05) those values obtained for the new, KTI absent mutants (A, B, and H).

RF at low frequency 13.5 MHz and moisture content 20.3% was performed to eliminate the harmful effect of biologically active trypsin inhibitors. For this work, the newly developed, high yielding conventional soybean variety (PK) and one of the investigated new, KTI absent soybean varieties (A) were selected and provided from the second year of cultivation for the RF heat treatment (75 °C, 100 °C, 110 °C for 50–90 s).

**Fig. 4:**
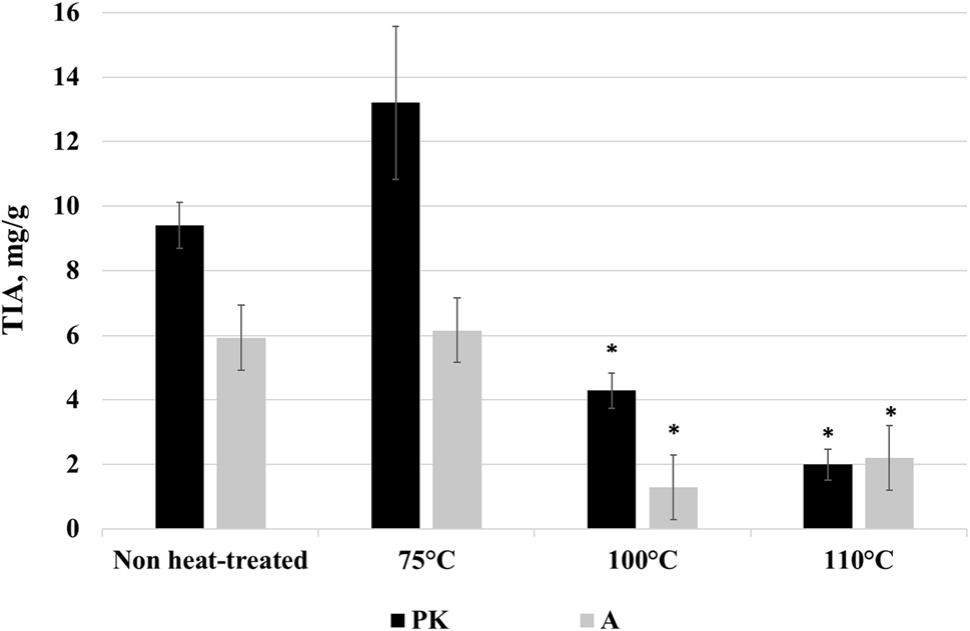
The TIA values (Mean ± SD) in intact *Glycine max.* (L) Merr. soybean varieties of PK (KTI mobility, Ti-a allele) and A (KTI absent mobility, ti allele) obtained from the second year of national cultivation influenced by RF heat treatments (75, 100, 110 °C obtained with 10 kW power on 13.5 MHz frequency, for 50–90 s). Values within each column with the * marking are significantly different from the non-treated conteurpart values (p < 0.05)

Effect of RF on the total TIA: TIA (mg/g) values measured (Fig. 4.) in the non-treated, crude samples (PK: 9.4 ± 0.71; A: 5.9 ± 0.69) were not changed meaningfully in either the traditional (PK75°C: 13.2 ± 2.38) or the KTI absent (A75°C: 6.2 ± 0.11) soybean samples, when the RF heat treatment was performed at 75 °C. At the same time a significant (p ≤ 0.05) decrease was observed in TIA values at the temperature ≥ 100 °C (PK100°C: 4.3 ± 0.55; PK110°C: 1.2 ± 0.48; A100°C: 1.3 ± 1.42; A110°C: 2.2 ± 0.60). However, the influence of the applied RF heat treatment was more effective in KTI absent soybean sample at 100°Cresulting a relative lower trypsin inhibitor activity, expressed as a percentage of intact soybean TIU activity (A100°C: 21.8%) in comparison with KTI mobility soybean sample (PK100°C: 45.6%), whereas such a decrease in PK occurred only at 110 °C(21.2%).

Recently, RF treatment was also employed at 27.12 MHz to inactivate trypsin inhibitors in intact soybean for minimising deleterious effects caused by conventional hot-air heating (Jiang et al., 2020). According to their results trypsin inhibitors were inactivated within 300 s and the relative trypsin inhibitory activity was reduced up to 10.6%. In addition, RF heating improved the functional and processing qualities of soybean products in contrast to the conventional thermal heat treatment where by achieving the similar inactivation rate, the functional properties significantly decreased because of the formation of greater aggregates. In their view, during conventional heat treatment, the surface temperature of soybeans rose sharply and the inhibitors located in the seed coat were rapidly inactivated. In contrary, the temperature in soybean seed coat and the internal tissues during the RF treatment initially was low and the relative inhibitory activity decreased slowly. By increasing the heating time, the internal temperature was able quickly reach the target temperature, sufficient for inactivation resulting a steep decrease in the relative inhibitory activity. Thus, RF treatment required less time and lower temperature to inactivate antinutritive components compared to conventional thermal treatment. Our results were comparable with those ones. Although our experiences were similar suggesting that RF heating has a great potential in effective inactivation of trypsin inhibitors, but the residual inhibitor activity is still representing an existing problem.

**Fig. 5:**
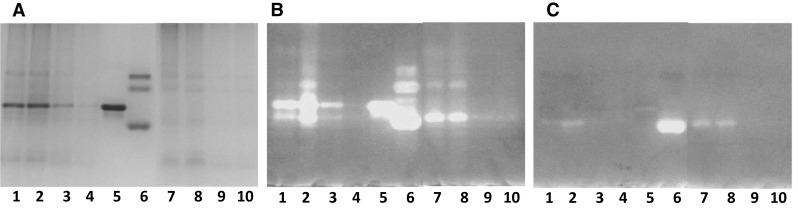
Patterns of separated soybean proteins [*Glycine max.* (L.) Merr.] in a newly developed conventional soybean variety (PK) and a KTI absent mobility type soybean variety **a** obtained from the second year of national cultivation, and influenced by RF heat treatments. The separation was performed in 6/12% native-PAGE **a**, where the trypsin **b** or the chymotrypsin **c** originated inhibitory activity were identified by negative enzyme staining. Lanes: 1. PK without RF treatment, 2. PK with 75 °C RF treatment, 3. PK with 100 °C RF treatment 4. PK with 110 °C RF treatment, 5. KTI 6. BBI, 7. A without RF treatment, 8. A with 75 °C RF treatment, 9. A with 100 °C RF treatment, 10. A with 110 °C RF treatment

Native-PAGE (Fig. 5) was combined with negative enzyme staining method to identify the residual KTI or BBI originated trypsin (Fig. 5b) and the BBI originated chymotrypsin (Fig. 5c) active bands after RF heat treatments. The effectiveness of heat inactivation of trypsin and chymotrypsin inhibitors is closely related to their structures (Xu et al. 2012). Purified KTI (one distinct band, Fig. 5, Lane 5) and BBI (three distinct bands, one for a monomer and two for the dimers, Fig. 5, Lane 6) were used as inside standards. In case of PK sample the KTI and BBI bands were definitely well-visible till 75 °C RF, but the intensity of these bands was effectively reduced at 100, 110 °C RF. (Fig. 5a, Lanes 1, 2, 3, 4 respectively). In case of KTI absent soybean variety (A) the BBI bands were still noticeable after 75 °C RF treatment, and remained gentle visible at 100, 110 °C RF as well (Fig. 5a, Lanes 7, 8, 9, 10, respectively).

The negative enzyme staining has shown that the residual trypsin inhibitor activity at 100 °C RF heat treatment mainly due to KTI-derived trypsin inhibitory activity in the PK soybean sample (Fig. 5b, Lane 3) and an effective reduction was only observed after 110 °C (Fig. 5b., Lane 4). Nevertheless, that the RF heat treatments ≥ 100 °C effectively reduced the intensity of the BBI-derived trypsin and chymotrypsin active bands in both types of soybean varieties (Fig. 5b., c, Lanes 3, 4, 9, 10) but the remaining trypsin active monomer bands may be responsible for the residual trypsin inhibitor activity.

Based on the above data at least 110 °C RF heat treatment seemed to be recommended for effective inactivation of trypsin inhibitors in conventional soybean seeds as PK, while a relatively lower temperature at 100 °C RF treatment was already effective for the KTI absent mobility type of soybean seeds as A. Lu et al. (2015) have found that KTI activity could be eliminated fast, while BBI could be eliminated only with stronger heat treatment. He et al. (2017) have obtained that the two subdomains of BBI in heat treated soybean milk were not equally heat stable and the trypsin binding site were more resistant to heat than chymotrypsin binding site. The heat inactivation was associated with conformational changes of BBI and the degradation of some amino acid residues (namely, cystine, serine and lysine). BBI did not tend to form intermolecular cross-links with another BBI, but did slightly with non-BBI proteins.

It is known that BBI is structurally and functionally resistant in extreme condition within the gut and can survive in bioactive form the action of proteolytic enzymes under simulated gastric and intestinal digestion (Clement and Argues 2014). Hajós et al. (1995) have demonstrated the presence of immunological reactive forms (5% of total ingested) of soybean BBI in the small intestine of rats. These data make BBI type of natural trypsin inhibitors attractive for further pharmacological and pre-clinical studies to be assessed their potential as colorectal chemo-preventive agents (Clement and Argues 2014).

**Fig. 6:**
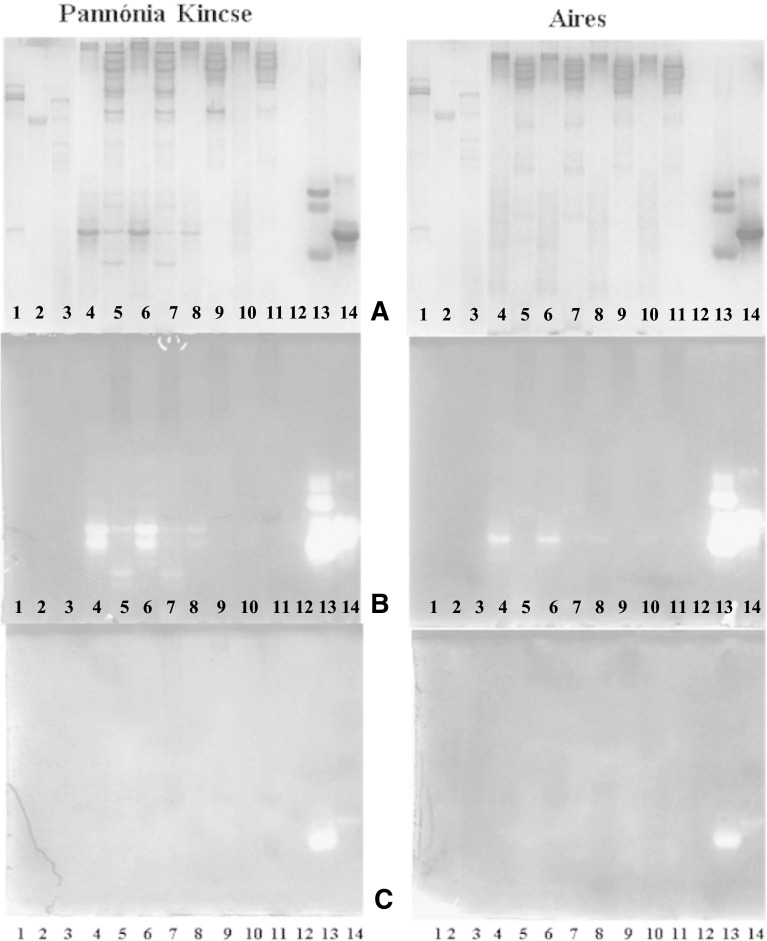
Patterns of separated soybean proteins [*Glycine max.* (L.) Merr.] after a static in vitro human digestion completed in simulated gastric and intestinal fluids. The samples of the conventional soybean seed (PK, KTI-a mobility) and the soybean seed with lower trypsin inhibitor activity (A, KTI absent mobility) obtained from the second year of national cultivation were assessed in native form and after RF heat treatments. The separation was performed in 6/12% native-PAGE **a**, where the trypsin **b** or the chymotrypsin **c** originated inhibitory activity were identified by negative enzyme staining. Lanes: 1. human salivary alpha-amylase, 2. porcine pepsin, 3. porcine pancreatin; 4–5. Sample without RF treatment, gastric and intestinal phases; 6–7. Sample with 75 °C RF treatment, gastric and intestinal phases, 8–9. Sample with 100 °C RF treatment, gastric and intestinal phases, 10–11. Samples with 110 °C RF treatment, gastric and intestinal phases, 12. -; 13. BBI; 14. KTI

A static in vitro digestion model was used to assess the gut resistance of the RF heat treated and the non-treated conventional (PK) and new soybean (A) varieties. To monitor the protein degradation process and functional activities of the residual trypsin inhibitors 6/12% native PAGE was applied (Fig. 6) followed by negative enzyme staining regarding to the KTI and BBI originated trypsin (Fig. 6b) and BBI originated chymotrypsin (Fig. 6c) inhibitory activities. Purified KTI (Lane 14) and BBI (Lane 13) and gut digestive enzymes such as human salivary alpha-amylase, porcine pepsin, porcine pancreatin (Lanes 1, 2, 3 respectively) were used as internal standards.

In native-PAGE gel single bands for KTI were still visible after the digestion in the simulated gastric fluids (SGF) for non-RF heat treated and RF heat treated PK soybean samples at 75, 100 °C, only disappeared at 110 °C, while the bands for BBI was only visible at 75 °C, then already faded at 100 °C, then at 110 °C too. (Fig. 6a, Lane 4, 6, 8, 10 respectively). At the same time KTI and BBI bands were unvisible in digested sample using simulated intestinal fluid (SIF) after 110 °C RF heat treatment (Fig. 6a, Lane 9, 11). While the protein bands corresponding to BBI protein in the KTI absent soybean (A) were faintly visible after digestion in the SGF until than the BBI bands were fragmented and very slightly seen in the gel after digestion in SIF (Fig. 6a, Lanes 5, 7, 9, 11).

The protein bands for conventional soybean (PK) stained for trypsin retained their inhibitory activity after the digestion in SGF (Fig. 6b, Lanes 4, 6, 8). After digestion in SIF the KTI originated trypsin active bands were still seen slightly in the gel for native and RF heat treated samples up to 75 °C, while the BBI originated trypsin active bands were fragmented not only in the RF heat treated samples at 75 °C but in the native samples as well (Fig. 6b, Lanes 5, 7). The trypsin active bands in SIF were no longer visible in the gel at higher temperature (≥ 100 °C) (Fig. 6b, Lanes 9, 11). The trypsin active, monomeric BBI bands after the SGF digestion of KTI absent soybean samples (A) gave a strong signal up to 75 °C and faintly visible signals at temperature higher than ≥ 100 °C. At the same time the dimers were not seen anymore in the gel. (Fig. 6b, Lanes 4, 6, 8, 10 respectively). It has been reported that soybean BBI is active at low pH in the presence of pepsin with no significant loss of inhibitory activity (Weder 1986). Although both the trypsin and chymotrypsin inhibitory sites of BBI was involved in this process, the structural rigidity provided by the disulphide bridges seems to exert a protective effect, avoiding an extensive proteolysis (Clement and Argues 2014). These findings were consistent with our results obtained for the investigated soybean samples digested in SGF.

At the same time, there were not observed any chymotrypsin inhibitory activity in soybean samples (PK, A) either after digestion in SGF nor in SIF (Fig. 6c, Lanes 4–11).

It was previously published that the BBI inhibitor binds to bovine duodenase by its anti-chymotrypsin site (Leu-Ser) and does not use its antitrypsin site (Lys-Ser), and therefore the BBI duodenase complex retains capacity for complete inhibition of trypsin (Gladysheva et al., 1999). Our experience was similar in case of SIF digested soybean samples as BBI must have bound to pancreatin by its anti-chymotrypsin site and does not use its anti-trypsin site, and therefore the BBI pancreatin complex retains capacity for complete inhibition of trypsin even after the applied RF heat treatments.

## Conclusion

As a result of an intensive breeding work in Hungary (Cereal Research Ltd., Szeged, Hungary) a significant increase in the volume of soybean production has occurred resulting the development of new, high-yielding (KTI mobility, Ti allele) soybean varieties and also the involvement of foreign varieties with low trypsin inhibitor content (KTI absent, ti allele) into the national cultivation resulting also the expansion of GM-free varieties.

In this study, the Kunitz (KTI) and the Bowman-Birk (BBI) types of trypsin inhibitors were characterized in *Glyicine max* (L) Merr. soybean seeds to learn about if an energy saving and gently RF heat treatment could be used as a good alternative for a conventional heat treatment resulting better digestibility of proteins.

Soybean seeds obtained from the two subsequent years of national cultivation at CR Ltd. were compared with well-characterized soybean varieties for KTI mobility having Ti-a or ti alleles. It was confirmed that the newly developed soybean variety (PK) where the two major trypsin inhibitors were present carried KTI mobility protein and had Ti-a allele as most of the European soybean varieties, whereas cultivars (A, H, B) with low trypsin inhibitor activities were lacking the KTI trypsin inhibitor and had ti allele. The presence of the KTI (Ti-a) or KTI null allele (ti) was also proven by using Satt228 DNA marker-specific PCR.

The bulk of soybean seeds were assessed for the temperature effect (75 °C, 100 °C, 110 °C) of low frequency (13.5 MHz) and short time (50–90 s) exposure radiofrequency (RF) heat treatments for the elimination of the residual trypsin inhibitor activity (TIA). It was found, that RF treatment at a lower temperature (75 °C) was not able to inactivate the residual BBI activities neither in the traditional or in the KTI absent samples but a significant decrease in TIA values was observed at the temperature ≥ 100 °C. At the same time at least 110 °C RF heat treatment seemed to be recommended for effective inactivation of trypsin inhibitors in conventional soybean seeds, while a relatively lower temperature at 100 °C RF treatment was already effective for the KTI absent mobility type of soybean seeds because of the residual KTI originated trypsin inhibitor activity. Although our experiences allowed to make a suggestion, that RF heating has a great potential in effective inactivation of trypsin inhibitors, the BBI originated residual inhibitor activity is still representing an existing problem.

We have shown that the trypsin inhibitors are still active in the presence of low pH and pepsin and both the trypsin and chymotrypsin inhibitory sites of BBI was involved in the digestion process. Probably, the RF heat treatment at temperature ≥ 100 °C and the digestion process can eliminate active inhibitors by affecting on their structural rigidity provided by the disulphide bridges.

On the above results we made a conclusion that both KTI mobility and KTI absent types of studied soybean varieties need to be treated at higher temperature (≥ 100 °C) using the applied short time and low frequency RF heat treatment to obtain better digestibility of proteins. In vitro human digestion model studies also showed that a residual BBI originated trypsin inhibitor activity was observed in the stomach even after RF treatment at 110 °C, whereas its chymotrypsin inhibitor activity in the small intestine was not detectable at all.

## Data Availability

Not applicable.

## Abbreviations

KTI: Kunitz trypsin inhibitor
BBI: Bowman-Birk trypsin inhibitor
TI: Trypsin inhibitor
TIA: Trypsin inhibitor activity
CIA: Chymotrypsin inhibitor activity
RF: Radiofrequency
PK: Pannónia Kincse (HU 149567)
A: Aires (IT 224)
H: Hilario
B: Bahia
DIC: Détente Instantane´e Contrȏlée (controlled instantaneous pressure drop technique)
HHP: High hydrostatic pressure
SPIs: Soy protein isolates
SIS: Società Italiana Sementi;
CR Ltd.: Cereal Research Non-Profit Ltd., Szeged, Hungary
PI: Plant Indroduction number in the USDA-ARS soybean germplasm collection
PCR: Polymerase chain reaction
DNA: Deoxyribonucleic acid
SSR: Simple sequence repeats
PAGE: Polyacrylamide gel electrophoresis
SD: Standard deviations
ANOVA: One-way analysis of variance
SSR: Simple sequence repeats
SIG: Simulated gastric fluid
SIF: Simulated intestinal fluid
Ti-a, Ti-b, Ti-c: *variant alleles of KTI* dominant gene (*Ti*)
ti: null KTI allele of the soybean resessive gene
L-BAPA: Na-benzoyl-DL-arginine 4-nitroanilide hydrochloride
DMSO: Dimetil sulfoxide
EDTA: Ethylenediaminetetraacetic acid
GM-free: genetically modified-free
